# Extruded Polystyrene Foams with Enhanced Insulation and Mechanical Properties by a Benzene-Trisamide-Based Additive

**DOI:** 10.3390/polym11020268

**Published:** 2019-02-05

**Authors:** Merve Aksit, Chunjing Zhao, Bastian Klose, Klaus Kreger, Hans-Werner Schmidt, Volker Altstädt

**Affiliations:** 1 Department of Polymer Engineering, University of Bayreuth, Universitaetsstrasse 30, 95447 Bayreuth, Germany; merve.aksit@uni-bayreuth.de (M.A.); chunjing.zhao@uni-bayreuth.de (C.Z.); 2 Macromolecular Chemistry I, University of Bayreuth, Universitaetsstrasse 30, 95447 Bayreuth, Germany; bastian.klose@uni-bayreuth.de (B.K.); klaus.kreger@uni-bayreuth.de (K.K.); 3 Bavarian Polymer Institute and Bayreuth Institute of Macromolecular Research, University of Bayreuth, Universitaetsstrasse 30, 95447 Bayreuth, Germany

**Keywords:** polystyrene foams, 1,3,5-benzene-trisamides, cell nucleation, foam extrusion, foam morphology, supramolecular additives, thermal insulation, compression properties

## Abstract

Low thermal conductivity and adequate mechanical strength are desired for extruded polystyrene foams when they are applied as insulation materials. In this study, we improved the thermal insulation behavior and mechanical properties of extruded polystyrene foams through morphology control with the foam nucleating agent 1,3,5-benzene-trisamide. Furthermore, the structure–property relationships of extruded polystyrene foams were established. Extruded polystyrene foams with selected concentrations of benzene-trisamide were used to evaluate the influence of cell size and foam density on the thermal conductivity. It was shown that the addition of benzene-trisamide reduces the thermal conductivity by up to 17%. An increase in foam density led to a higher compression modulus of the foams. With 0.2 wt % benzene-trisamide, the compression modulus increased by a factor of 4 from 11.7 ± 2.7 MPa for the neat polystyrene (PS) to 46.3 ± 4.3 MPa with 0.2 wt % benzene-trisamide. The increase in modulus was found to follow a power law relationship with respect to the foam density. Furthermore, the compression moduli were normalized by the foam density in order to evaluate the effect of benzene-trisamide alone. A 0.2 wt % benzene-trisamide increased the normalized compression modulus by about 23%, which could be attributed to the additional stress contribution of nanofibers, and might also retard the face stretching and edge bending of the foams.

## 1. Introduction

With the development of technology and society worldwide, the energy demand is increasing constantly, while fossil energy resources are becoming increasingly short. In the European Union, the total energy consumption of buildings accounts for more than 40%. Additionally, the CO_2_ emitted by buildings and constructions corresponds to almost a quarter of the global CO_2_ emissions [1]. However, over 60% of the energy is wasted by heat loss through building elements such as walls, roofs, floors, and windows [2,3,4,5]. Therefore, the thermal insulation of buildings is of high environmental importance. Among the commonly used thermal insulation materials for building applications such as glass, stone wool, and polymer foams, polymer foams accounted for a share of 41% in 2015 and are reported to exhibit the fastest growth rate during the next 10 years [6]. In this context, extruded polystyrene (XPS) foams play a significant role for thermal insulation applications given their ease of foaming, low price, and distinguished thermal insulating and mechanical properties [7,8,9,10]. Therefore, XPS foams have been extensively applied as insulating material in floor panels and in basement outer walls of buildings [11,12].

In contrast to macrocellular XPS foams with a cell size larger than 100 µm, microcellular XPS foams with a cell size smaller than 10 µm possessing the same density offer improved mechanical and insulation properties due to their microcellular morphology [13]. Therefore, most studies have been conducted based on cell size reduction in order to achieve structure–property optimization [8,14,15,16]. Recently, a novel class of nucleating agents, namely 1,3,5-benzene-trisamide (BTA), was investigated with regard to its effect on the morphology control of polypropylene (PP) foams [17,18]. Depending on the concentration and process conditions, BTA can be completely dissolved in polymer melt. Upon cooling, the dissolved BTA molecules can self-assemble into nanofibers which can act as a heterogeneous nucleating agent. Typical issues such as agglomeration, which is associated with the use of inorganic additives [19], can be avoided, while a high surface area for cell nucleation is provided. For semi-crystalline PP, BTAs can act not only as foam nucleating agents [17,18] but also as nucleating agents for the polymer crystallization [20]. Therefore, by applying BTA to amorphous polystyrene (PS), the nucleation of polymer crystallization is excluded, and thus only the cell nucleation effect is present. The principle cell nucleation capability of BTAs in PS was shown in the patent application from Clariant [21] and the recent paper from our group, in which the influence of BTA concentration on foam morphology was revealed [22].

Here, we demonstrate that XPS foams containing BTA can lead to improved thermal insulation behavior and mechanical properties when compared to neat XPS foams, which are important for the application of insulation panels in buildings. The optimization in foam properties are discussed at different BTA concentrations and the structure–property relationships of the XPS foams are established.

## 2. Materials and Methods 

### 2.1. Materials

Commercial PS (trade name: PS168N) from INEOS STYROLUTION (Frankfurt am Main, Germany), of which the molecular weight (M_w_) is 340 kg/mol and the polydispersity index (PDI) is 2.3, was used. BTA (chemical name: 1,3,5-Tris(2,2-dimethylpropionylamino) benzene, trade name: Irgaclear XT386) from BASF SE (Ludwigshafen, Germany) was used as a foam nucleating agent at concentrations of 0.1 wt %, 0.2 wt % and 0.5 wt %.

XPS foams used in this study were produced by a tandem extrusion line from Dr. Collin GmbH (twin-screw extruder with a 25-mm screw and L/D 42; single-screw extruder with a 45-mm screw and L/D 30) equipped with a slit die with a 0.6-mm gap and a 30-mm width. The processing parameters for foam extrusion were set as 260 °C, from 113 °C to 118 °C, and 126 °C for the melt temperature in the first extruder, the melt temperature in the second extruder, and the die temperature, respectively. The mixture of 4 wt % of CO_2_ and 3 wt % of ethanol was used as the physical blowing agent [14]. The cell size and cell density were determined by the software Image J using SEM micrographs of the XPS foams. At least 70 cells were taken into account to determine the average cell size and cell density. The obtained morphological properties and densities of the neat XPS foam and XPS foams with BTA are summarized in Table 1. It should be noticed that 0.2 wt % BTA reduced cell size most efficiently, while the higher concentration of 0.5 wt % BTA increased the cell size slightly due to its incomplete solubility at this processing condition, resulting in larger aggregates in the PS matrix.

### 2.2. Thermal Conductivity

The thermal conductivities of the foam samples were measured by the heat flow meter LaserComp FOX 50 from TA Instruments. Foam samples were cut into cylinders with a diameter of 60 mm and thicknesses (*L*) between 3 mm and 8 mm depending on the extruded foam thickness. The samples were positioned between two temperature-controlled plates. These plates established a temperature difference (Δ*T*) of 10 °C across the samples by setting the upper plate as 30 °C, while the lower plate was set as 20 °C. The resulting heat flux (*Q*/*A*) through samples was measured by two proprietary thin film heat flux transducers. Thermal conductivities (λ) were calculated according to Equation (1):

(1) $$\lambda =\frac{Q}{A}\frac{L}{\Delta T}.$$

At least five samples from each foam at different positions were measured and average values of the thermal conductivities were determined.

### 2.3. Mechanical Properties

The compression moduli of extruded PS foams were measured by a Universal Test Machine (Z050, ZwickRoell GmbH & Co. KG, Ulm, Germany) based on ISO 844. Samples for the compression tests were prepared by cutting foams into cylinders with a diameter of 10 mm and a length of 10 mm. The compression loads were applied perpendicular to the extrusion direction of the foam samples. The compression strain was limited to 30%, which was sufficient to characterize the modulus and plateau stress values for each sample. The test speed was 1 mm/min with a 0.5-N preload to ensure full contact between the sample surfaces and plates of the test machine. At least five samples of each XPS with and without BTA were tested.

## 3. Results and Discussion

### 3.1. Effect of BTA on the Thermal Conductivity of XPS Foams

**Figure 1:**
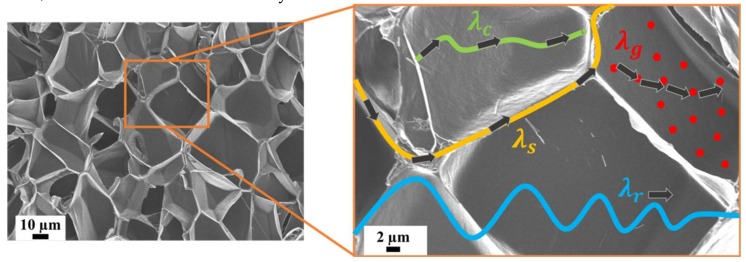
Schematic representation of heat transfers in foams.

Thermal conductivity is crucial when considering XPS foams applied as insulation panels in buildings and constructions. To elaborate the complicated mechanism of thermal insulation improvements, the different thermal contributions need to be discussed individually. In foams, it is assumed that the total thermal conductivity ($\lambda_{t}$) can be described by four different contributions, as expressed in Equation (2):

(2) $$\lambda_{t}\;=\;\lambda_{c}+\lambda_{s}+\lambda_{g}+\lambda_{r}$$

where $\lambda_{c}$ represents the thermal convection between neighbouring foam cells, which can be neglected as all extruded foam samples had closed cells and cell sizes smaller than 4 mm [23]; $\lambda_{s}$ is the thermal conduction along the solid phase, namely cell walls and cell struts; $\lambda_{g}$ is contributed by the thermal conduction across the cells by the impulse transfer of gas molecules to the cell walls and struts; and $\lambda_{r}$ is the thermal radiation term, which is caused by electromagnetic radiation emitted by all surfaces [12]. When radiative energy passes through the foam, it undergoes (i) adsorption by solid; (ii) reflection at the interface; and (iii) transmission [24]. Thermal radiation only plays a significant role for low density foams (<40 kg/m^3^) [25]. Moreover, $\lambda_{r}$ is a temperature-dependent term showing an increase by a function of 3 with the increasing average temperature of the inside and outside temperature [26]. A schematic representation of the heat transfer mechanisms in foams is shown in Figure 1. Each term is influenced by various factors such as foam density, cell size, cell wall and strut thickness, and the thermal conductivity of solid BTA.

**Figure 2:**
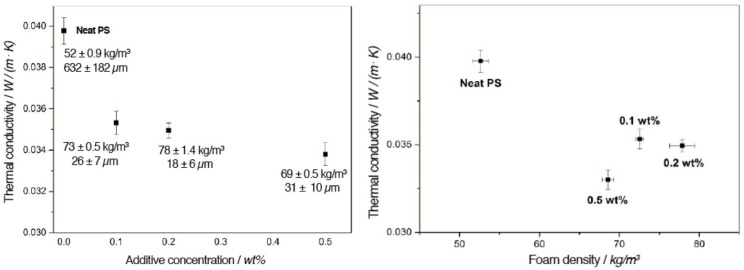
Thermal conductivity of XPS foams including foam density and mean cell size with increasing additive concentration (**left**) and with increasing foam density (**right**).

$\lambda_{s}$ is mainly determined by the intrinsic thermal conductivity of solid materials (PS and BTA) and the amount of their content in the foams. A lower foam density leads to a decrease in $\lambda_{s}$ due to the reduced contribution from the solid matrix [27]. On the other hand, the contribution from gas molecules, i.e., air, in foam cells ($\lambda_{g}$) is influenced by the cell size. As the cell size decreases, the energy transfer by air in the cells is significantly reduced. However, a cell size reduction is achieved at the expense of an increase in foam density, which in turn causes a higher $\lambda_{s}$. Therefore, there is an optimal foam density for the lowest thermal conductivity. Figure 2 exhibits the change in the thermal conductivity of the XPS foams with the increasing additive concentration and foam density.

As illustrated in Figure 2 (left), thermal conductivities of XPS foams with BTA at all concentrations were significantly lower than that of neat XPS foam (0.040 W/(m·K)), indicating an improved thermal insulation performance. The largest decrease of about 17% in thermal conductivity was achieved for foams containing 0.5 wt % BTA. Moreover, XPS foams with 0.1 wt % and 0.2 wt % BTA had similar thermal conductivity due to their similar foam densities and foam cell sizes. We found that foams with 0.1 wt % and 0.2 wt % BTA still led to a reduction in the thermal conductivity by about 11% and 12%, respectively. As shown in Figure 2 (right), foams with 0.5 wt % BTA exhibited the lowest thermal conductivity, which can be attributed to the optimal compromise between cell size reduction and an increase in foam density.

### 3.2. Effect of BTA on the Compression Modulus of XPS Foams

**Figure 3:**
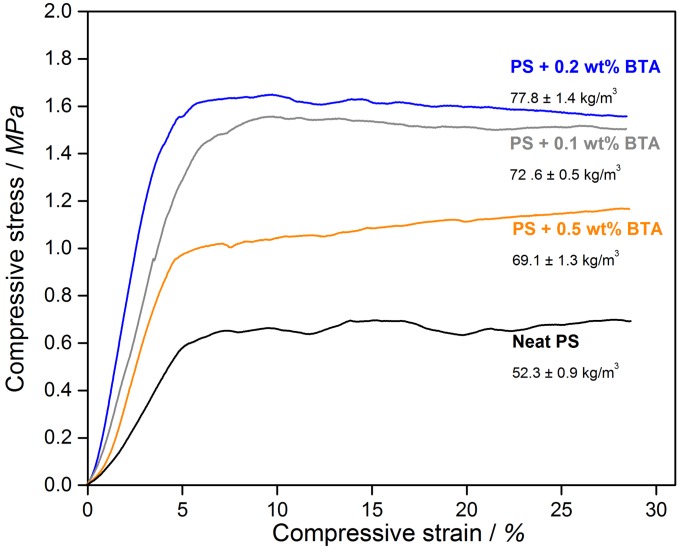
Compressive stress–strain diagram of the curves for neat XPS and XPS foams with various BTA concentrations.

Aside from enhanced thermal insulation properties, XPS foams should exhibit a sufficient compression modulus (typically in the range of 6.5 to 25 MPa [28]) to meet the application requirements as insulation for floor panels and outer walls of basements in buildings and constructions where the foams are mainly subjected to compression loads. The mechanical properties of the PS foams are strongly influenced by foam density as well as a variety of other factors such as the intrinsic reinforcing effect of additives, the orientation of fibrillar additives, the cell opening effect, and the gas pressure inside the closed-cell foams. Figure 3 shows the representative stress–strain curves of the extruded neat PS and PS with 0.1 wt %, 0.2 wt %, and 0.5 wt % BTA.

**Figure 4:**
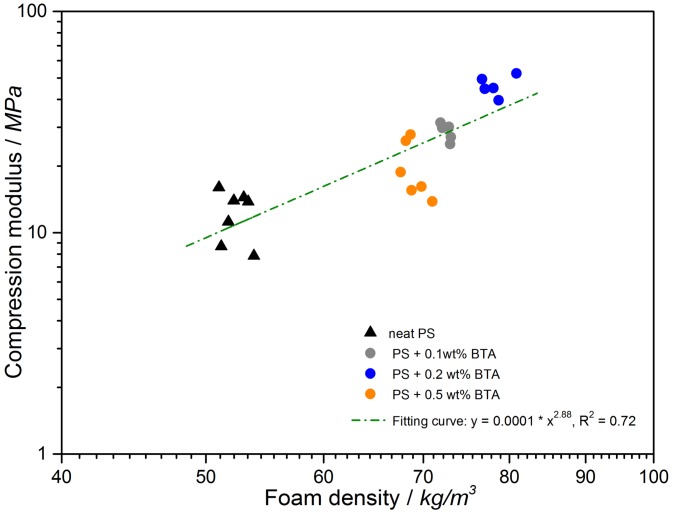
Change in the compression modulus independent of foam density.

According to Figure 3, all of the curves possessed an initial linear elasticity region where the elastic bending of the cell walls and face stretching took place. The slope of the tangent at the linear elasticity region provided the compression modulus. A plateau region in the compression stress was observed beyond the linear-elastic regime. Elastic buckling of the unaligned struts led to the plastic collapse of the cells and stress at 10% to 20% of deformation corresponded to the plastic collapse stress [29]. It can be clearly seen (Figure 4) that there was an increase in the compression modulus as well as the plastic collapse stress of the BTA-containing foams when compared to those of the neat XPS foams. The highest plastic collapse stress and the largest slope of the linear elasticity region corresponding to the highest compression modulus were achieved by the XPS foam with 0.2 wt % BTA. The improvement in the compression moduli and plastic collapse stresses might be due to an increase in foam density with the addition of BTA and the effect of nanofibers on cell walls and struts. In order to validate the correlation between the foam density and the compression modulus, compression moduli were plotted with respect to the foam density in a logarithmic scale in Figure 4.

**Figure 5:**
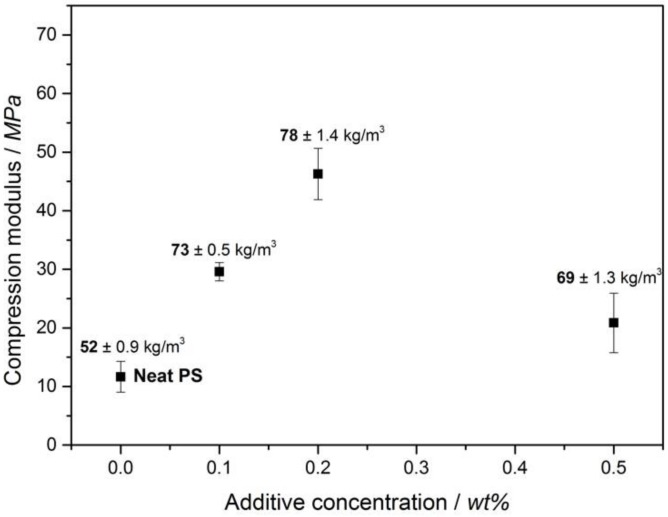
Compression moduli of XPS foams independent of BTA concentration.

According to Figure 4, with increasing foam density, the compression moduli became higher, proving that foam density plays a significant role on the compression modulus. By increasing the fraction of the solid matrix in foams in both the cell walls and cell struts, the compression modulus increased due to the increased foam density. This behavior was also explained by the Gibson–Ashby model [29]. According to the model, the stiffness of a closed-cell foam results from three contributions. The first component is the cell struts and cell wall edge bending stiffness, which determines the elastic modulus. The second component is the cell wall elastic buckling, which causes elastic collapse. The final component is the internal gas pressure of the closed cells, which only plays a minor role in the atmospheric pressure and small deformations. Thus, the sum of the first two components can be expressed by Equation (3):

(3) $$\frac{E_{f}}{E_{s}}=\phi^{2}{\left(\frac{\rho_{f}}{\rho_{s}}\right)}^{2}+\left(1-\phi \right)\frac{\rho_{f}}{\rho_{s}}$$

where *E*_𝑓_ is the elastic modulus of the foam; *E*_𝑠_ is the elastic modulus of the solid material; 𝜌_𝑓_ is the foam density; 𝜌_𝑠_ is the solid polymer density; *ϕ* is the fraction of polymer contained in the cell struts, and 1 − *ϕ* is the solid fraction in the cell walls. The simplified Equation (4) can be yielded from Equation (3), showing a power law relationship describing the functional dependence of the modulus on the foam density.

(4) $$E\alpha \rho^{n}$$

where *n* is the density exponent of the foam. For bulk materials, the density exponent is 1, suggesting a linear relationship between the modulus and solid material density. Theoretically, the value of *n* should be between 1 and 2 for closed-cell foams [30]. A fitting curve, which is shown in Figure 5, was obtained by fitting at least five values from each sample to a power function. The power law equation described the relationship between the compression moduli and foam density very well, except for foams with 0.2 wt % BTA. For these foams, the compressive moduli increase was sharper, leading to a coefficient of determination, *R*^2^, of 0.72 and a density exponent of 2.88, which was higher than the expected value of 2 for closed foams. This might be due to an additional stress contribution induced by BTA nanofibers with diameters of 200 to 600 nm, depending on the concentration. In our previous study [22], we observed that BTA nanofibers located in cell walls and struts of XPS foams contributed further to the improvement in the compression moduli of foams. The most significant increase in the compression modulus at the BTA concentration of 0.2 wt % will be discussed further. Figure 5 exhibits the influence of BTA concentration on the compression modulus. In general, a significant improvement in the compression moduli of the XPS foams with BTA in comparison with that of the neat XPS foam was found. In the best case, the compression modulus increased from 11.7 ± 2.7 MPa for the neat PS to 46.3 ± 4.3 MPa with 0.2 wt % BTA. However, a further increase in the BTA concentration to 0.5 wt % led to a decrease in foam density as well as a decrease in the compression modulus due to the stress concentration points in foam that resulted from aggregates induced by the incomplete solubility of BTA [22]. These imperfections in the porous non-straight and misaligned struts might lead to weakened mechanical properties.

However, it is well known that foam density has a significant effect on the compression modulus [29]. In order to eliminate this effect and better understand the sole influence of BTA on the compression modulus of the foams, the normalized compression moduli at 52.3 kg/m^3^ were calculated. The normalization calculations were conducted using Equation (5), which was derived from Equation (4) [31]:

(5) $$E_{normalized\;}=\;E_{measured\;}*{\left(\frac{\rho_{reference}}{\rho_{measured}}\right)}^{n}$$

where $E_{normalized}$ is the normalized compression modulus; $E_{measured}$ is the measured modulus of the foam sample; $\rho_{reference}$ is the foam density of the reference (neat XPS) foam (52.3 kg/m^3^); $\rho_{measured}$ is the measured density of the sample; and *n* is the slope of the log compression modulus versus log foam density graph (Figure 5), which was 2.88.

**Figure 6:**
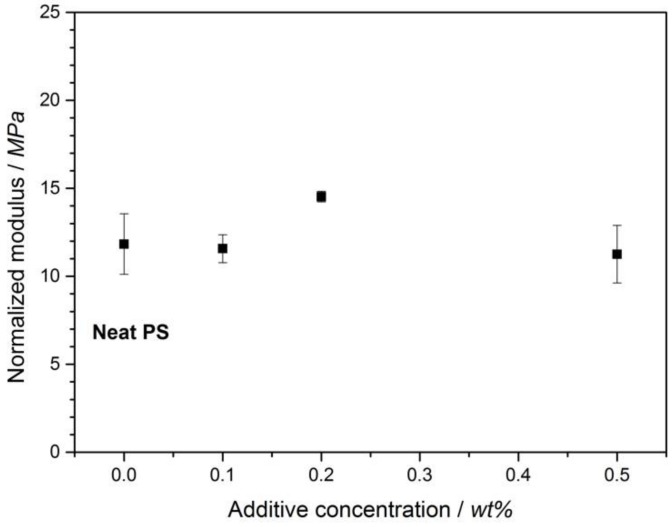
Normalized compression moduli of the XPS foams with different concentrations of BTA compared to the neat XPS foam.

Figure 6 depicts the density and normalized compression moduli of the neat XPS foam and XPS foams with different BTA concentrations at a foam density of 52.3 kg/m^3^.

Figure 6 illustrates the normalized compression moduli at the reference density of 52.3 kg/m^3^ and determines the influence of BTA solely on the mechanical properties of PS foams. The highest increase in the normalized compression modulus of about 23% when compared to the neat XPS was obtained at the BTA concentration of 0.2 wt %. While the neat XPS foams exhibited a relatively large standard deviation of the normalized modulus, the XPS with 0.2 wt % BTA possessed the minimum standard deviation. This might be correlated with the increase in the uniformity of the foam morphology induced by BTAs. The addition of 0.1 wt % BTA was not enough to improve the modulus of the XPS foam significantly. The normalized modulus at 0.5 wt % BTA decreased by about 12% when compared to that of the neat XPS foam. The reduction in the compression modulus and increase in standard deviation in the modulus could be attributed to the agglomeration of non-dissolved BTA due to the incomplete solubility of the additive at concentrations higher than 0.2 wt %, which is the solubility limit in PS at this foaming condition. Based on the improved compression moduli of the XPS foams with 0.2 wt % BTA, we attributed this to the nanofibers with a high aspect ratio on the cell walls and struts [22], providing an additional stress contribution to the compression modulus. These may also retard the face stretching and edge bending, leading to increased buckling resistance and better mechanical properties of the foam. A similar reinforcing effect of BTA nanofibers on compression mechanical properties has already been shown for PP by Mörl et al. [18].

## 4. Conclusions

In this study, the improved thermal insulation behavior and mechanical properties of XPS foams containing BTA were investigated. Furthermore, the structure–property relationships of a neat XPS foam and XPS foams with 0.1 wt %, 0.2 wt %, and 0.5 wt % of BTA as the foam nucleating agent were established with regard to thermal insulation and mechanical performance. Although the cell size reduction achieved through 0.5 wt % BTA was less effective than that achieved with 0.1 wt % and 0.2 wt % BTA due to its partial solubility in PS, the lowest thermal conductivity of 0.033 W/(m·K) was obtained with 0.5 wt % BTA. The 17% reduction in thermal conductivity, when compared to that of the neat XPS foam, was due to the optimum compromise between the competing effects from cell size and foam density. While the smaller cell sizes reduced the gas thermal conductivity ($\lambda_{g}$), the higher foam density increased the solid thermal conductivity ($\lambda_{s}$). On the other hand, XPS foams with 0.2 wt % BTA showed the highest compression modulus of 46.3 MPa (seven times the reinforcement when compared with that of the neat XPS foam), which was attributed to the highest foam density of 78 kg/m^3^. Additionally, the enhancement of the normalized compression modulus of the XPS foam with 0.2 wt % BTA showed that the fibrillar nanofibers of BTA positively influenced the mechanical properties of the foams.

## Figures and Tables

**Table 1:** Density and morphological properties of extruded polystyrene (XPS) foams.

Sample	Foam density (kg/m^3^)	Cell size (µm)	Cell density (cells/cm^3^)
Neat XPS	52.3 ± 0.9	632 ± 182	2.7 × 10^3^
XPS + 0.1 wt % BTA	72.6 ± 0.5	26 ± 7	5.6 × 10^7^
XPS + 0.2 wt % BTA	77.8 ± 1.4	18 ± 6	1.5 × 10^8^
XPS + 0.5 wt % BTA	69.1 ± 1.3	31 ± 10	3.1 × 10^7^
